# Intraoperative Wound Lavage System for Deep Neck Infection: A Retrospective Cohort Study

**DOI:** 10.1055/s-0042-1758717

**Published:** 2023-10-06

**Authors:** Soraya Abdul-Hadi, Francis Beauchamp Perez, Jeamarie Pascual-Marrero

**Affiliations:** 1 Division of Otolaryngology, Head and Neck Surgery, School of Medicine, University of Puerto Rico, San Juan, Puerto Rico

**Keywords:** debridement, infection, lavage, neck, surgical wound, deep neck infection

## Abstract

**Introduction** Multiple solutions are currently used to cleanse a deep neck infection (DNI), and a variety of devices are available to deliver wound irrigation solutions. An essential difference between these devices is the pressure that the irrigation solution exerts over the wound tissue.

**Objective** To compare low-pressure and high-pressure irrigation delivery systems for wound cleansing in DNI.

**Methods** we designed a retrospective cohort study and reviewed the medical records of patients operated on due to DNI from June 2016 to December 2017 at our institution. One cohort included patients treated with an intraoperative irrigation method that exerts low pressure over the irrigated tissue, and the other cohort, to a system capable of generating higher pressure. The Pearson Chi-squared test was used to analyze the data.

**Results** A total of 42 patients whose ages ranged from 16 months to 72 years were included. The low-pressure irrigation system was used in 18 patients, and the high-pressure system was used in 24 patients. No statistical differences were observed regarding the irrigation methods, the complexity of the DNI, and the overall outcomes.

**Conclusions** The present is the first study in which low- and high-pressure systems for wound lavage were evaluated in the treatment of DNI. When comparing these methods, we did not find one to be superior to the other; however, the additional cost associated with the high-pressure devices may not justify their in head and neck procedures.

## Introduction

The most common etiologies of deep neck infections (DNIs) are odontogenic infections, usually polymicrobial in nature, due to poor oral hygiene, followed by contiguous extension of tonsillar/pharyngeal infections. 1

The mechanism by which these infections spread is through lymphatic extension, hematogenous dissemination, or direct extension. 2 These infections may ascend or descend through the deep neck fascia planes, increasing morbidity and mortality. 3 Airway compromise, descending mediastinitis, and necrotizing fasciitis are among the severe complications. 4

Appropriate patient care requires knowledge of the location and extent of the infection. Therefore, characterization with detailed imaging studies is crucial. Contrast-enhanced computed tomography (CT) scans are the gold standard for diagnosis. Regarding management, aggressive monitoring and control of the airway is the priority, followed by appropriate antibiotic coverage. If surgical intervention is warranted, intraoral and/or extraoral incision and drainage should be performed. The incision provides the means for drainage and for cleansing of the pocket collection. Currently, there are various cleaning solutions available and different mechanical methods to deliver the wound irrigation solutions.

To date, no study has shown the superiority of any of the cleansing solutions used in the head and neck. The most common solutions currently in use are normal saline solution, and solutions containing iodine and antibiotics. 5 However, there is no available data to determine if there is any significant difference between the intraoperative mechanical delivery method used to clean after incision and drainage.

The irrigation pressure in pounds per square inch (psi) that is applied over the tissue depends on the mechanical method used. Some studies suggest that methods that exert an irrigation pressure lower than 4 psi are not sufficient for adequate cleaning. One example of a low-pressure system is the bulb syringe, which exerts pressure ranging from of 0 psi to 1 psi. Moreover, multiple studies recommend the use of an irrigation method that generates pressure between 4 psi and 15 psi for adequate cleansing. Nonetheless, this recommendation is based on data that has been exported from studies that compare different mechanical methods for wound irrigation outside the head and neck. 6

Based on these studies, multiple devices that exert different amounts of pressure have been developed for wound irrigation, such as the Irrisept (Irrimax Corporation, Lawrenceville, GA, United States), a manual device that exerts pressure ranging from 7 psi to 8 psi. 7

Irrisept contains 0.05% of chlorhexidine gluconate in sterile water. Chlorhexidine gluconate is known as an antiseptic agent with broad antimicrobial action against Gram-positive and Gram-negative organisms, anaerobes, aerobes, and yeasts. Nonetheless, the low concentration of chlorhexidine gluconate in Irrisept acts solely as a preservative for the product. 8 9 10

To our knowledge, there is a lack of data on the use of different mechanical methods for the intraoperative lavage of DNIs. 11 It is well established that the use of a device that exerts between 4 psi and 15 psi has an additional benefit for wound irrigation when compared with conventional methods. 6 However, the use of these devices in the head and neck region has not been documented.

The main objective of the present study was to compare two different mechanical methods for intraoperative neck wound lavage. The goal was to create one cohort composed of patients treated with a low-pressure system (bulb syringe) and compare it to a second cohort, composed of patients in whom the lavage was performed with Irrisept, a system capable to generate higher pressure.

## Methods

The present study was reviewed and approved by the Institutional Review Board (approval number B1660118).

We performed a retrospective cross-sectional analysis of patients with a diagnosis of DNI managed at our institution from June 2016 to December 2017. We chose to start the analysis in 2016 because this is when the Irrisept irrigation system was introduced for DNIs at our institution. Using the Electronic Medical Record (EMR) database, data on the patient population was obtained with the use of applicable International Classification of Diseases (ICD) 9 ^th^ and 10 ^th^ edition codes for head and neck infections.

The inclusion criteria were patients diagnosed with deep neck space infection by physical examination and/or CT scan, and those whose surgical intervention was solely performed by the Otolaryngology–Head and Neck Service of our institution from 2016 to 2017.

The exclusion criteria were diagnosis of peritonsillar abscess, surgical intervention at other institutions, and treatment discontinuation against medical advice.

A total of 42 medical records were reviewed. For each patient, the following data was collected: demographics, comorbidities, smoking history, history of ethanol or drug use, symptomatology in history of present illness, previous formation of neck abscess, current location of the DNI, CT findings pertinent to the location of the DNI, history of antibiotic use, type of surgery performed (transcervical approach, transoral approach or both), irrigation method used during surgery (low- or high-pressure device), airway management, drain placement, amount of steroids required after surgery, the need for reintervention in the operating room, length of hospital stay, and postoperative complications.

The study personnel extracted the pertinent data from each record using a collection instrument. The database was developed using Microsoft Excel (Microsoft Corp., Redmond, WA, United States) spreadsheets and stored in computer network files of the Division of Otolaryngology–Head and Neck Surgery in a secure location. A master database was then established, and a non-identifiable ID number was assigned to each patient. This was all performed in accordance with regulations of the Health Insurance Portability and Accountability Act (HIPAA).

Two study cohorts were created according to the mechanical irrigation method used during surgery: the first was composed of patients treated with an intraoperative low-pressure (0 psi to 1 psi) irrigation method (bulb syringe), and the second, of patients treated with a high-pressure system (Irrisept).

For the statistical analysis, a univariate analysis was performed using the sample size (n) and the relative frequencies (%). The data analyzed included the therapeutic outcome and surgical treatment. Pearson Chi-squared tests were used, with values of *p* < 0.05 considered statistically significant.

## Results

Within the study period, 42 patients underwent surgery due to DNI. Most of the patients were male (76%) and older than 18 years of age (83%). The most common comorbidities were hypertension, immunocompromised state, and diabetes mellitus. Most of the sample had never smoked (64%), and the most common symptoms presented by them were dysphagia, neck pain, and odynophagia ( Table 1 ).

**Table 1:** Demographics of the study participants

Patient demographics	n (%)*
**Gender**	
Male	32 (76%)
Female	10 (24%)
**Age**	
> 18 years old	35 (83%)
< 18 years old	7 (17%)
**Comorbidities**	
Hypertension	12 (29%)
Immunocompromised (cancer, obesity, daily steroid use, end-stage renal disease, hepatitis C virus)	8 (19%)
Diabetes mellitus	6 (14%)
Intravenous drug abuser	2 (4%)
**Smoking status**	
Never smoked	27 (64%)
Smoker Unknown	8 (19%) 7 (17%)
**Presenting symptoms**	
Dysphagia	29 (69%)
Neck pain	25 (60%)
Odynophagia	22 (52%)
Sore throat	15 (36%)
Change in voice (hoarseness, muffled voice)	10 (24%)
Trismus	10 (24%)
Neck swelling	7 (17%)
Shortness of Breath	6 (14%)

Note: *Total *n* = 42.

In most of the cases ( *n* = 10), multiple neck spaces were affected. The parapharyngeal space was most commonly affected ( *n* = 10), followed by the retropharyngeal space ( *n* = 8). Abscess formation was observed at the anterior compartment of the neck in 3 patients, and at the masticator space in another 3 patients; 6 subjects had necrotizing fasciitis; 1 had an abscess at level 5 (left side); and 1 had a submental abscess.

The patients were hospitalized for a mean of 15 days (range: 3 to 103 days). All patients underwent incision, drainage, and cleansing of their DNI in the operating room. All patients received intravenous antibiotics on admission.

The Pearson Chi-squared test was performed to examine whether the initial surgery and treatments were independent ( Table 2 ). Three surgical approaches were used in the initial surgery: transcervical, transoral, and the combined approach. For wound irrigation, two methods were compared: the bulb syringe, which is classified as a device with low-output pressure, the Irrisept, a high-output pressure system.

**Table 2:** Comparison of initial surgical approach and irrigation system

Initial surgery	Irrigation with highpressure (psi) device: n (%)	Irrigation with low-pressure (psi) device: n (%)	*p* -value
Transcervical	23 (96%)	12 (67%)	**0.034**
Both approaches	1 (4%)	3 (17%)	
Transoral	0 (0%)	3 (17%)	

The comparison between the surgical approach and irrigation method used ( Table 2 ) revealed that the results of the Chi-squared test of independence were statistically significant ( *p* = 0.034), suggesting that the initial surgery and treatment (irrigation method) are related to one another. This means that for both irrigation systems (high- and low-pressure), the approach most commonly performed in the initial surgery was the transcervical (96.0% and 67.0% respectively), followed by the combined (4.0% and 17.0% respectively) and transoral approaches (0% and 17.0% respectively).

The association regarding the requirement for intraoperative tracheostomy and previous use of antibiotics with the irrigation system used is shown in Tables 3 and 4 respectively. No statistically significant difference between the high- and low-pressure groups was found ( *p* = 0.35 and 0.21, respectively). Furthermore, Table 5 presents the associations involving the postoperative requirement of a high dose of steroids, the need for reintervention, and the hospital length of stay (LOS) with the treatments. We found no statistically significant difference between the high- and low-pressure groups in terms of the need for steroids ( *p* = 0.22), for reintervention ( *p* = 1.00), and the hospital LOS ( *p* = 0.78). The same ratio of intra-operative drain placement was found between both treatment groups.

**Table 3:** Comparison of incidence of tracheostomy placement and irrigation system

Tracheostomy	Irrigation with high-pressure (psi) device: n (%)	Irrigation with low-pressure (psi) device: n (%)	*p* -value
Yes	14 (58%)	7 (39%)	0.35
No	10 (42%)	11 (61%)	

**Table 4:** Comparison of previous use of antibiotics and irrigation system

Previous use of antibiotics	Irrigation with high-pressure (psi) device: n (%)	Irrigation with low-pressure (psi) device: n (%)	*p* -value
Yes	8 (33%)	10 (56%)	0.21
No	16 (67%)	8 (44%)	

**Table 5:** Association between postoperative high dose of steroids, surgical reintervention, hospital length of stay, and irrigation system

Postoperative high dose of steroids	Irrigation with high-pressure (psi) device	Irrigation with low-pressure (psi) device	*p* -value
Yes	11 (46%)	12 (67%)	**0.22**
No	13 (54%)	6 (33%)	
**Need for reoperation**			
Yes	5 (21%)	3 (17%)	**1.00**
No	19 (79%)	15 (83%)	
**Length of stay**			
0–7 days	9 (38%)	9 (50%)	**0.78**
8–14 days	7 (29%)	4 (22%)	
> 14 days	8 (33%)	5 (28%)	

The postoperative complications (endpoints) reported were laryngeal edema ( *n* = 3), pneumonia ( *n* = 4), septic shock ( *n* = 1), empyema ( *n* = 2), scar hypertrophy ( *n* = 3), mediastinitis ( *n* = 1), and pneumomediastinum ( *n* = 1). They were compared between the two treatment groups, and no statistically significant difference was found ( Table 6 ).

**Table 6:** Association between complications and irrigation system

Complications	Irrigation with high-pressure (psi) device: n (%)	Irrigation with low-pressure (psi) device: n (%)	*p* -value
Yes	9 (38.0%)	9 (50.0%)	**0.42**
No	15 (62.0%)	9 (50.0%)	

## Discussion

As seen in the present study, peritonsillar, parapharyngeal, and retropharyngeal collections are the most common sites of infection. 12 Patients with peritonsillar abscesses were excluded from our analysis because most of them do not require intervention in the operating room. From our data, the parapharyngeal was the deep neck space most commonly affected, followed by the retropharyngeal space.

The anatomic limitations and the direction for the spread of these DNIs are demarcated by multiple layers of cervical fascia that form at least 11 deep neck spaces. 13 The morbidity and mortality associated with DNI and abscess are decreasing due to improvements in airway management, availability of CT imaging, and earlier presentation and diagnosis. 2

Some of the risk factors for the development of deep neck abscess are poor oral hygiene leading to odontogenic infection and immunocompromised state, such as that caused by diabetes mellitus. It has been reported 8 that other risk factors for the development of DNI include tonsillitis and smoking. Nonetheless, most of our sample had never smoked.

Incision and drainage are the mainstays of the surgical therapy. 11 However, reinfection is a potential complication, and proper wound cleaning, debridement, and postoperative care should be provided. There are multiple irrigation methods available for proper wound cleansing after incision and drainage. Some of the standard irrigation methods currently used for head and neck infections are normal saline solution and solutions containing iodine and antibiotics.

To enhance wound cleansing, multiple methods to deliver the irrigation solution have been described in the literature. However, to date, there is no consensus to define what is a high- and a low-pressure device. Nonetheless, the current literature favors irrigation pressure between 5 psi and 15 psi. Lower pressure is associated with inadequate removal of debris, and higher pressure, with tissue trauma. 6 14 15 Yet, to the extent of our knowledge, there are no studies on the effect of the mechanical methods of the irrigation systems in the head and neck region.

In the present study, we compared the irrigation method with a bulb syringe, which exerts an output pressure of 0 psi to 1 psi to the Irrisept system, which exerts 7 psi 8 psi of pressure over the tissue.

After a retrospective review, we found no statistical differences regarding the irrigation methods and the need for tracheostomy, for a postoperative high dose of steroids, or the use of antibiotics prior to incision and drainage. With these results, we can extrapolate that there is no difference in the complexity of the DNI between the groups. Additionally, we found no statistically significant difference involving the hospital LOS and the need for surgical reintervention between the two cohorts.

Moreover, DNIs are characterized by their rapid evolution and spread. Consequently, patients can suffer from severe complications, such as acute edema of the larynx with respiratory obstruction, mediastinitis, necrotizing fasciitis, empyema, pericarditis, and jugular vein thrombosis. 1 2 Based on this data, we collected information on the development of these complications in the postoperative period. Nonetheless, when comparing irrigation methods and the incidence of these complications, the rates appear to be similar for both groups ( Table 6 ). A possible explanation is that both irrigation methods have the same efficacy.

Published data suggests that there is no difference in the cleansing efficacy between low- and high-pressure irrigation systems. Moreover, some studies 16 17 report that pulsatile lavage irrigation and high-pressure systems can impair the wound healing process. However, in the present study, the Irrisept system was a safe and viable alternative for irrigation of DNIs after incision and drainage because no harmful or adverse events were reported after its use.

Nonetheless, the present study contains several major limitations. One of the notable limitations is its retrospective nature and small sample size. In addition, there was a difference in the baseline characteristics of both groups. Six of the included patients had necrotizing fasciitis, which is known to be a distinct disease entity, characterized by a more aggressive course. Moreover, pediatric and adult DNIs are somewhat distinct entities. Pediatric patients tend to experience a more favorable natural course of the disease when compared with adult patients.

Additionally, the choice of intraoperative irrigation method represents a bias because we opted to use the high-pressure delivery system when the patient had a more serious infection such as in the cases of necrotizing fasciitis. Also, more than 50% of the pediatric patients were part of the low-pressure group. These differences in baseline characteristics between the cohorts could have affected the outcomes of the present study.

Therefore, it is reasonable to conclude that we did not find significant statistical differences between the treatment groups because the high-pressure group had a higher patient load with a more aggressive infectious process, and the low-pressure group had a higher load of patients with favorable outcomes (pediatric population). This distinction could explain why we did not observe better outcomes in the high-pressure group.

Moreover, data on the use of irrigation outside of the operating room through drains and the drain removal timeline were missing, which has also added a source of bias to the present study.

Due to the aforementioned bias, the strength of the conclusions we can draw is limited, and the external validity of the study is not high. Nonetheless, the present study is very valuable because it opens the possibility of conducting of future studies at our institution and/or at other academic centers.

## Conclusion

The present study is valuable because it is the first to evaluate the difference between surgical wound irrigation systems in the head and neck. At this point, we suggest that the rates of reoperation, postoperative outcomes, and complications were similar regardless of the irrigation pressure used. This finding indicates that the low-pressure system is an acceptable, low-cost alternative for the irrigation of DNIs. Nonetheless, a more robust study, such as a prospective randomized control trial, is necessary to assess if there is a difference between the irrigation method used in the head and neck for surgical wound cleansing.
